# Editorial Comment: Management of sphincter insufficiency in patients with neurogenic bladder and bladder augmentation

**DOI:** 10.1590/S1677-5538.IBJU.2023.06.03

**Published:** 2024-02-07

**Authors:** Andrés G. Alfieri, Marcio A. Averbeck

**Affiliations:** 1 Hospital Italiano de Buenos Aires Buenos Aires Argentina Hospital Italiano de Buenos Aires, Buenos Aires, Argentina;; 2 Hospital Moinhos de Vento Unidade de Videourodinâmica Porto Alegre RS Brasil Chefe de Neuro-Urologia, Unidade de Videourodinâmica, Hospital Moinhos de Vento. Porto Alegre, RS, Brasil

Seppo Taskinen 1, Eija Mäkelä 2, Niklas Pakkasjärvi 2

**Pediatr Surg Int. 2023 Jun 28;39(1):221.**

**DOI: 10.1007/S00383-023-05506-x| ACCESS: 37378684**

## COMMENT

This is a retrospective study over 29 years at a single institution, which evaluated the need and effectiveness of bladder neck procedures (BNPs) in patients with neurogenic lower urinary tract dysfunction (NLUTD) undergoing bladder augmentation. Patients were evaluated preoperatively with urodynamics, urinary tract ultrasound, renal function tests, and cystoscopy. In total, 87 patients were included in the analysis, with a median follow-up of 10.7 years. Urinary continence was achieved by 64 patients (74%), while occasional episodes of urinary incontinence were reported by 19 (22%) patients, and 4 patients (5%) remained incontinent (pad usage). Overall, 37 patients (43%) were treated by BNPs, including bladder neck injections (BNI) in 28 patients, fascial sling operation in 14 patients, and bladder neck closure (BNC) in five females. BNI and fascial sling procedures had comparable results for both sexes. Full continence was achieved in 10/28 (36%) patients with one or repeat BNIs and 9/14 (64%) with sling operation. The four female patients requiring BNC achieved urinary continence. In this cohort, myelomeningocele was the most common diagnosis and incontinence was mostly caused by neurogenic sphincter underactivity.

The primary goals for management of patients with NLUTD include protecting the upper urinary tract, achieving a safe situation in the lower urinary tract, providing a reservoir of sufficient volume and improving quality of life (1). In high-risk patients, bladder augmentation techniques have an established role despite inherent perioperative morbidity. Taskinen et al considered BNPs in two circumstances: if the patients remained incontinent despite an adequate size and low-pressure bladder, or concomitantly with bladder augmentation when sphincter underactivity was evident. Timing for BNPs may be challenging due to diagnostic uncertainties related to borderline urodynamic findings and low adherence to intermittent catheterization.
